# Phosphorylation of SU(VAR)3–9 by the Chromosomal Kinase JIL-1

**DOI:** 10.1371/journal.pone.0010042

**Published:** 2010-04-06

**Authors:** Joern Boeke, Catherine Regnard, Weili Cai, Jørgen Johansen, Kristen M. Johansen, Peter B. Becker, Axel Imhof

**Affiliations:** 1 Adolf-Butenandt Institute and Munich Center of Integrated Protein Science (CIPS), Ludwig Maximilians University of Munich, Munich, Germany; 2 Department of Biochemistry, Biophysics & Molecular Biology, Iowa State University, Ames, Iowa, United States of America; Institute of Genetics and Molecular and Cellular Biology, France

## Abstract

The histone methyltransferase SU(VAR)3–9 plays an important role in the formation of heterochromatin within the eukaryotic nucleus. Several studies have shown that the formation of condensed chromatin is highly regulated during development, suggesting that SU(VAR)3–9's activity is regulated as well. However, no mechanism by which this may be achieved has been reported so far. As we and others had shown previously that the N-terminus of SU(VAR)3–9 plays an important role for its activity, we purified interaction partners from *Drosophila* embryo nuclear extract using as bait a GST fusion protein containing the SU(VAR)3–9 N-terminus. Among several other proteins known to bind Su(VAR)3–9 we isolated the chromosomal kinase JIL-1 as a strong interactor. We show that SU(VAR)3–9 is a substrate for JIL-1 *in vitro* as well as *in vivo* and map the site of phosphorylation. These findings may provide a molecular explanation for the observed genetic interaction between SU(VAR)3–9 and JIL-1.

## Introduction

The modifications of specific residues within the histone N-termini serve as a marking system that contributes to the establishment and maintenance of distinct chromatin structures [1], [2], [3], [4]. Modified amino acids are recognized by chromatin-binding factors that distinguish between differentially modified histones [5], [6], [7] and are involved in the organization of chromatin [8], [9]. Proteins that interact with modified histones can also be regulated themselves by posttranslational modifications [10]. For example HP1, the well-known binding factor for histone H3 methylated at lysine 9 (H3K9me), is phosphorylated at multiple sites [11], [12], [13]. These phosphorylations appear to be necessary for its biological function to set up a characteristic heterochromatic structure [11], [14]. Surprisingly little is known about the regulation of the enzymes that catalyze the formation of the posttranslational modifications. The histone methyltransferases Suv39H1 and ENX2 are phosphorylated *in vivo* [15], [16], but the exact position, the regulation or the biological function of the modification has so far remained elusive. Only recently several groups showed that distinct signaling pathways could indeed modulate methyltransferase function [17], [18], [19]. Besides phosporylation, histone methyltransferases are able to catalyze their own auto-methylation, which has been suggested to regulate their activity [20]. However, the biological function of this modification remains unclear.

One of the best-characterized chromatin structures *in vivo* is the pericentric constitutive heterochromatin [21]. In a popular model system for monitoring the repressive effect of heterochromatin, active genes are juxtaposed to pericentric heterochromatin by a large chromosomal inversion. Thereby the expression of these genes becomes sensitive to repression by near-by heterochromatin [22]. This phenomenon called “position effect variegation” (PEV) allowed the genetic isolation of suppressors and enhancers of heterochromatin mediated repression [23]. Until now over 50 different suppressor (*Su(var)*) or enhancer (*E(var)*) mutants were isolated in several laboratories, but only a few of them have been characterized in detail. Among these are the known chromatin binding factor HP1 (SU(VAR)2–5) [24] and the histone methyltransferase SU(VAR)3–9 [25]. Another interesting enzyme that was isolated as a suppressor of position effect variegation is the dual kinase JIL-1/SU(VAR)3-1 [26], [27]. JIL-1 had initially been identified as a kinase implicated in dosage compensation of *Drosophila* [28]. Besides this rather specific function, mutation of *JIL-1* also has major consequences on global chromosome structure as it leads to deranged chromosomes [29].

Although several factors involved in heterochromatin formation have been defined for some time, we are far from understanding the principles that allow a coordination of heterochromatin formation with other physiological events such as the cell cycle or external signals. Here we show that two factors that are involved in forming specific chromatin structures, the histone methyltransferase SU(VAR)3–9 and the kinase JIL-1, physically interact. Furthermore, the chromosomal kinase JIL-1 is able to phosphorylate SU(VAR)3–9 at a specific residue within the N-terminus, a region that is important for its function. Our data together with the recent discovery that *JIL-1* genetically interacts with *Su(var)3–9* but not with *Su(var)2–5* [30] suggest that the phosphorylation of SU(VAR)3–9 may play a role in fine-tuning its ability to mediate heterochromatin formation and spreading.

## Materials and Methods

### Plasmids

GST- and His-tagged dSU(VAR)3–9 and dSU(VAR)3–9 deletion constructs were cloned and expressed in bacteria as described earlier [31]. Constructs for GAL4-DBD fusions of SU(VAR)3–9 and for the expression of SU(VAR)3–9 in a baculovirus system were cloned by inserting PCR fragments into pActGAL4 or pFastBac HTB (details available on request). Two different expression constructs were used for the expression of recombinant JIL-1 kinase from Sf9 cells. For GST-JIL(40–1207), JIL-1 coding sequence from the pGEX 4T3 clone [32] was cloned into pAcGHLT-A (BD, Biosciences). For JIL(1–1207) expression, the full length coding sequence was PCR-amplified from the EST clone AT19088 (BDGP) and cloned into pCRII-TOPO (Invitrogen), which was used for *in vitro* translation of the JIL-1. The full length sequence was cloned into pVL1392 (Invitrogen) with an N-terminal flag-tag for expression in Sf9 cells. For the generation of point mutants of SU(VAR)3–9 and for the Flag-Jil-1^D392A^ mutant, which is catalytically inactive, mutagenesis was carried out using the QuikChange Site-Directed Mutagenesis Kit (Stratagene) (Details are available on request).

### Affinity purification of proteins binding to the SU(VAR)3–9 N-terminus

GST and GST-SU(VAR)3–9NT (aa 1–152) were expressed in *Escherichia coli* BL21 and individually bound to GSTrap FF columns (GE Healthcare). Parallel columns A and B were coupled with GST and GST SU(VAR)3–9NT respectively, and a *Drosophila* nuclear extract from 0–12 hour embryos was loaded. After a washing step (200 mM NaCl, 20 mM Tris-HCl (pH 8.0), 1 mM EDTA, 0.5% Nonidet P-40), a step elution (250, 500 and 750 mM) of the bound proteins was conducted on an ÄKTA-FPLC system (GE Healthcare). Fractions were analyzed for bound proteins by SDS-PAGE followed by silver staining and/or Western Blot.

### Antibodies

Polyclonal rabbit anti-S191ph antibodies were raised against the peptide KRRRSS(p)CVGAP (Eurogentec) and subsequently affinity-purified to enrich for the phospho-specific antibodies. Monoclonal rat antibodies against SU(VAR)3–9 were described in [33].

### GST pull-down of *in vitro* translated proteins

GST and GST fusion proteins were expressed in *E. coli* BL21. GST pull-downs were carried out essentially as described earlier [34]. Bacteria were induced with 0.2 mM isopropyl-D-thiogalactopyranoside (IPTG) for 3 h at 37°C. Recombinant proteins were purified with glutathione-sepharose beads (GE Healthcare) and analyzed by SDS-PAGE to normalize protein amounts. Equivalent amounts of GST fusion proteins were incubated with [^35^S]-methionine-labeled proteins, produced by the T7/T3 TNT-coupled transcription/translation system (Promega) in 200 µl of binding buffer (100 mM NaCl, 20 mM Tris-HCl (pH 8.0), 1 mM EDTA, 0.5% Nonidet P-40, 5 µg of ethidium bromide, 100 µg of bovine serum albumin (BSA)). After 0.5 h of incubation at room temperature, the beads were washed 5 times with 1 ml of binding buffer without ethidium bromide and BSA. The bound proteins were eluted with SDS sample buffer, separated by SDS-PAGE, and visualized by autoradiography.

### Cell Culture and Transfection

*Drosophila* Schneider cells (SL2) were grown in Schneider's *Drosophila* medium (Gibco) +10% fetal calf serum and transfected using the Effectene Transfection Reagent (Qiagen) according to the manufacturer's instructions. Luciferase reporter assays were performed essentially as described earlier [35]. To activate the basal transcription of the pG_5_DE_5_-tkluc reporter expression plasmids for *dorsal* and *twist* were cotransfected with the indicated GAL-fusion proteins and a plasmid carrying the *Renilla* luciferase gene under the control of the tk-promotor to normalize transfection efficiency. After 48 hours the transfected cells were harvested, lysed and analyzed for luciferase expression using the Dual-Luciferase Reporter Assay System (Promega). Expression of the Gal-fusion proteins were monitored by Western blotting by using an anti-GAL antibody (N19, Santa Cruz Biotechnology).

### Kinase assays

Recombinant and purified JIL-1 kinase (GST- or flag-tagged) were mixed with the indicated substrate proteins in kinase buffer (20 mM Hepes pH 7.6, 1 mM MgCl_2_, 1 mM EGTA, 5 mM NaF, 0.1 mM Na_3_VO_4_, 10 µM ATP) + [^32^P]-labeled γATP and incubated for 30 min at 26°C. The reactions were stopped by addition of sample buffer and 20–100% of the reactions were analyzed by SDS-PAGE. After Coomassie staining of the gels, to visualize equal protein amounts, the SDS-gels were dried and analyzed on a Phosphoimager.

### Immunocytochemistry of co-targeted LacI-Su(var)3–9 and LacI-JIL-1 to polytene chromososomes

The DNA-binding domain of the lacI repressor from *E. coli* was fused to the NH_2_-terminus of the full-length *Su(var)3–9* cDNA inserted in the pUAST vector. LacI-Su(var)3–9 pUAST fly lines were generated by standard P-element transformation (BestGene, Inc.). The LacI-JIL-1 and LacI-JIL-1 catalytically inactive pUAST lines have been described [36] and the Lac operator insertion line P11.3 was described in [37], [38]. Third instar larvae co-expressing LacI-Su(var)3–9 together with LacI-JIL-1 or with LacI-JIL-1 catalytically inactive protein were generated by standard genetic crosses with expression driven by the *Sgs3-GAL4* driver (obtained from the Bloomington Stock Center). Acid-free polytene squash preparations were made and labeled with antibody as described [39]. Primary antibodies used include rabbit anti-Su(var)3–9 S191ph (this study), rabbit anti-Su(var)3–9 (gift of Dr. G. Reuter), mouse anti-lacI (Upstate Biotechnology), rabbit anti-JIL-1 [40], chicken anti-JIL-1 [32], and anti-JIL-1 mAb 5C9 [32]. DNA was visualized by staining with Hoechst 33258 (Molecular Probes) in PBS.

## Results

**Figure 1:**
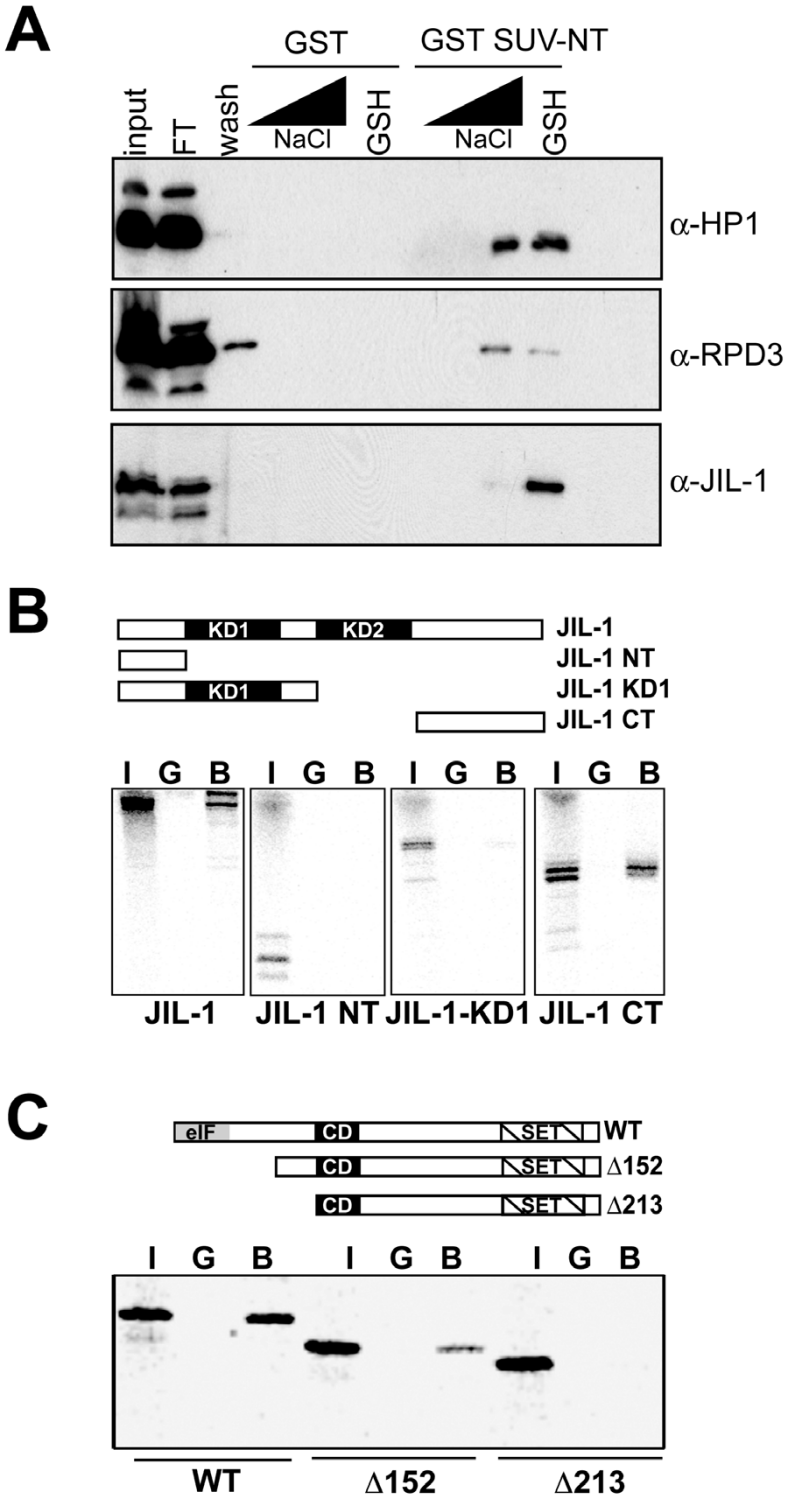
Identification of the chromosomal kinase JIL-1 as a novel interaction partner of SU(VAR)3–9. (**A**) Western Blot analysis of the eluates (250, 500, 750 mM Nacl and reduced Glutathion, GSH) from the indicated columns, identifies JIL-1 as a novel interactor of SU(VAR)3–9. 15 µl of each fraction were separated by SDS-PAGE and subjected to Western Blot analysis using the indicated antibodies (FT =  flow through). (**B**) The JIL-1 C-terminus mediates the interaction with SU(VAR)3–9. *Top*: Schematic representation of the JIL-1 derivatives used for the *in vitro* translation reaction indicating the characteristic domain structure of JIL-1 (KD1/KD2  =  kinase domain 1 and 2). *Bottom*: GST pull-downs using bacterially expressed GST or GST SU(VAR)3–9NT together with the indicated JIL-1 constructs. (**C**) A region within the N-terminus of SU(VAR)3–9 interacts with JIL-1. *Top*: Schematic representation of the SU(VAR)3–9 constructs used for the *in vitro* translation reaction indicating the characteristic domain structure of the protein (*e*IF =  homology region to the eukaryotic translation initiation factor 2; CD =  chromodomain; SET: SET domain). *Bottom*: GST pull-downs using bacterial expressed GST or GST JIL-1 full length together with the indicated recombinant SU(VAR)3–9 proteins. The GST fusion proteins were expressed in *E. coli*, purified, and incubated with *in vitro* translated, ^35^S-labeled JIL-1 or SU(VAR)3–9 (and deletions thereof). I = 5% input used for the precipitation; G =  GST; B =  Bound.

The N-terminus of SU(VAR)3–9 plays a crucial role in regulating the enzyme's function *in vitro* [31], [41]. As we had previously shown that it mediates an intramolecular protein-protein interaction [31], we wondered whether additional proteins might interact with this domain thereby regulating the function of the methyltransferase. Therefore we expressed the N-terminus of SU(VAR)3–9 as a GST-fusion protein in bacteria and used this as a bait to isolate specific interactors from nuclear extracts of *Drosophila* 0–12 hr embryos. Testing candidate factors we found that among several other known interactors such as HP1 or RPD3, the chromosomal kinase JIL-1 also interacted specifically with the N-terminus of SU(VAR)3–9 (Figure 1A). We did not observe an interaction with the endogenous SU(VAR)3–9 suggesting that either the concentration of endogenous SU(VAR)3–9 is too low or that it is entirely involved in interactions with other factors.

### SU(VAR)3–9 interacts with JIL-1

To test whether the interaction between JIL-1 and SU(VAR)3–9 is mediated by a direct protein-protein contact, we performed GST-pull down experiments using *in vitro* translated JIL-1 deletion mutants and the N-terminal region of SU(VAR)3–9 fused to GST (Figure 1B) or *in vitro* translated SU(VAR)3–9 and a GST-JIL-1 fusion protein (Figure 1C). This allowed us not only to verify a direct interaction between SU(VAR)3–9 and JIL-1 but also to map the interaction domains between SU(VAR)3–9 and JIL-1 to the N-terminus of SU(VAR)3–9 and the C-terminus of JIL-1. This is particularly interesting as mutants lacking the C-terminus of JIL-1 act as suppressors of position effect variegation [27].

**Figure 2:**
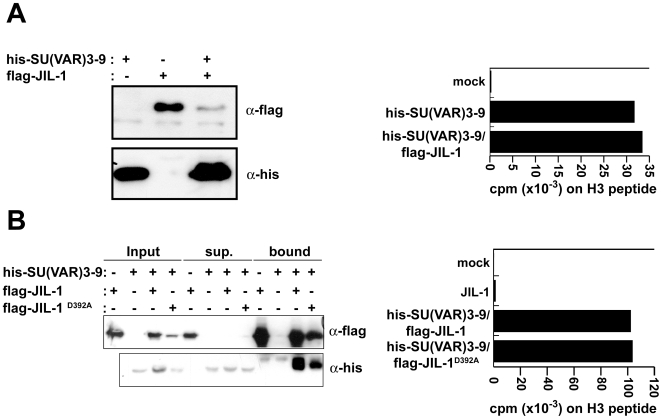
SU(VAR)3–9 and JIL-1 interact *in vivo*. (**A**) Coexpression of JIL-1 and SU(VAR)3–9 in SF9 cells using a baculoviral expression system. *left*: Western Blot of whole cell Sf9 extract. Proteins were detected using the indicated antibodies. *right:* Histone methyltransferase assay after co-infection of SF9 cells with flag-JIL-1 and his-SU(VAR)3–9 followed by affinity purification on a Talon™ metal affinity resin. (**B**) Precipitation of JIL-1 from an SF9 cell extract co-purifies active SU(VAR)3–9. *left*: Western Blot analysis of flag-coimmunoprecipitations using SF9 whole cell extract after co-infection with the indicated viruses. After the Co-IP flag M2-beads were washed and eluted with the flag peptide. 10% of the eluates were separated by SDS PAGE and analyzed by Western blotting using specific antibodies. *right:* Histone methyltransferase assay after co-infection of SF9 cells with flag-JIL-1, a flag-JIL^D392A^ and his-SU(VAR)3–9 followed by affinity purification on a flag M2 affinity resin.

Next we wanted to find out whether the two proteins can also form a complex within living cells. We co-expressed JIL-1 and SU(VAR)3–9 using a baculoviral expression system (Figure 2A). As JIL-1 carried a flag- and SU(VAR)3–9 a his-tag we could verify the formation of a complex using affinity purification. SU(VAR)3–9 forms a complex with JIL-1 as it was purified using a flag affinity resin only when flag-JIL-1 was co-expressed (Figure 2B). This is independent of JIL-1 kinase activity as a mutant that can no longer phosphorylate histones (JIL-1^D392A^) [36] is still able to interact with SU(VAR)3–9 (Figure 2B). As JIL-1 and SU(VAR)3–9 interact genetically as well as biochemically we wondered whether the interaction changed the ability of SU(VAR)3–9 to methylate H3 on lysine 9 (K9). Although the baculovirally expressed protein is more active than the one expressed in bacteria (data not shown), we did not observe a substantial influence of JIL-1 co-expression on SU(VAR)3–9's ability to methylate H3 (Figure 2C).

### JIL-1 phosphorylates the N-terminus of SU(VAR)3–9

**Figure 3:**
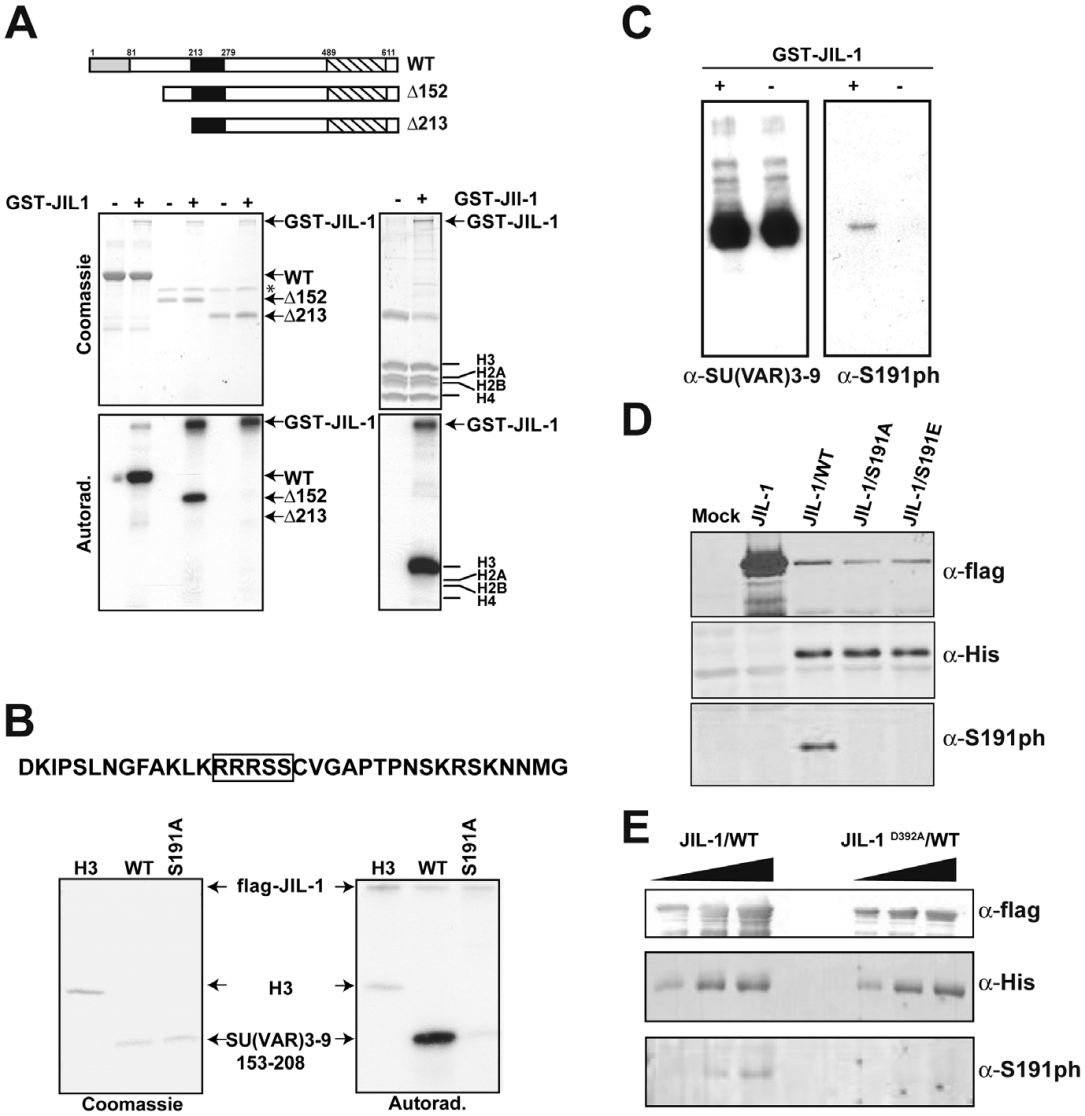
SU(VAR)3–9 is a target for phosphorylation by the chromosomal kinase JIL-1. (**A**) JIL-1 phosphorylates the N-terminus of SU(VAR)3–9 *in vitro*. *top*: schematic representation of the SU(VAR)3–9 constructs used for the *in vitro* kinase reaction (WT =  SU(VAR)3–9; Δ152/Δ213 =  N-terminal deletions of 152 or 213 amino acids respectively). Bottom *left*: *in vitro* kinase reactions with recombinant and purified GST-JIL-1 (expressed in SF9 cells using a baculoviral system) and bacterial expressed, affinity purified his-SU(VAR)3–9. Bottom *right*: As a control reaction *Drosophila* histones were analyzed in a kinase assay (+/− GST-JIL-1). The band labeled with an asterisk corresponds to BSA, which was included in the purification procedure. (**B**) The serine 191 of SU(VAR)3–9 (S191) is the major phosphorylation site. *top*: Amino acid sequence of the putative phosphorylation domain as determined by the kinase assays in panel (**A**). The boxed sequence motif indicates a homology to a cAMP kinase consensus site. *bottom*: *in vitro* kinase assay with recombinant and purified flag-JIL-1 and bacterial expressed, affinity purified GST-SU(VAR)3–9 fusion proteins containing the putative phosphorylation domain (PD: aa 153–208) or a mutation of S191 to A. As a control reaction recombinant *Drosophila* histone H3 was assayed in comparison. (**C**) Specificity of the *anti*-SU(VAR)3–9-phospho antibody (α-S191ph). Recombinant his-SU(VAR)3–9 was subjected to an *in vitro* kinase assay with or without GST-JIL-1 (+/−). After stopping the reaction with SDS-loading buffer, the reactions were separated by SDS-PAGE followed by Western blotting using α-S191ph and an antibody against SU(VAR)3–9, respectively. (**D**) SU(VAR)3–9 WT but not S191A mutant proteins can be phosphorylated by JIL-1. (**E**) An inactive mutant of JIL-1 (JIL-1^D392A^) cannot phosphorylate SU(VAR)3–9. SF9 infected with increasing amounts of the indicated viruses (JIL-1 =  flag JIL-1; JIL-1^D392A^ =  catalytically inactive form of flagJIL-1 WT  =  hisSU(VAR)3–9 wild type protein).

It has been proposed that the kinase activity of JIL-1 is crucial for its *in vivo* function [26], [28] and that JIL-1 acts in the same genetic pathway as SU(VAR)3–9 [30], [42]. We therefore tested whether SU(VAR)3–9 might be a direct substrate of the JIL-1 kinase. *In vitro* assays showed that JIL-1 can indeed phosphorylate SU(VAR)3–9 within its N-terminus (Figure 3A). By mutational analysis we were able to narrowed down the site of phosphorylation to amino acids 153–208 (Figure 3A). In order to precisely map the phosphorylation site within SU(VAR)3–9 we cleaved the *in vitro* phosphorylated peptide from the GST moiety with thrombin and analyzed the phosphorylation product using MALDI-TOF mass spectrometry (data not shown). Thereby, we could narrow down the phosphorylation site to amino acids 174–208. This peptide contains a moderately conserved recognition site for MSK/RSK kinases, to which JIL-1 belongs (Figure 3B). Mutation of the serine 191 to alanine (S191A) leads to a protein that is no longer phosphorylated by JIL-1 (Figure 3B) suggesting that S191 is the major residue that is phosphorylated by JIL-1.

In order to study the regulation of SU(VAR)3–9 we generated a phospho S191 (S191ph) specific antibody that recognizes SU(VAR)3–9 only after phosphorylation by JIL-1 (Figure 3C). Using this antibody, we tested whether JIL-1 could phosphorylate SU(VAR)3–9 *in vivo*. As the expression levels of SU(VAR)3–9 are extremely low in SL2 cells as well as in *Drosophila* embryos, we had to resort to an over-expression system. We over-expressed wild type as well as mutated SU(VAR)3–9 in SF9 cells using recombinant baculoviruses. Mutation of S191 to either A or E did not affect the interaction of SU(VAR)3–9 with JIL-1 but prevented phosphorylation (Figure 3D). Moreover, the phosphorylation was dependent on active JIL-1 as the catalytically inactive JIL-1^D392A^ still interacted with SU(VAR)3–9 but was no longer able to phosphorylate it (Figure 3E).

### Phosphorylation of SU(VAR)3–9 does not affect its ability to repress transcription

**Figure 4:**
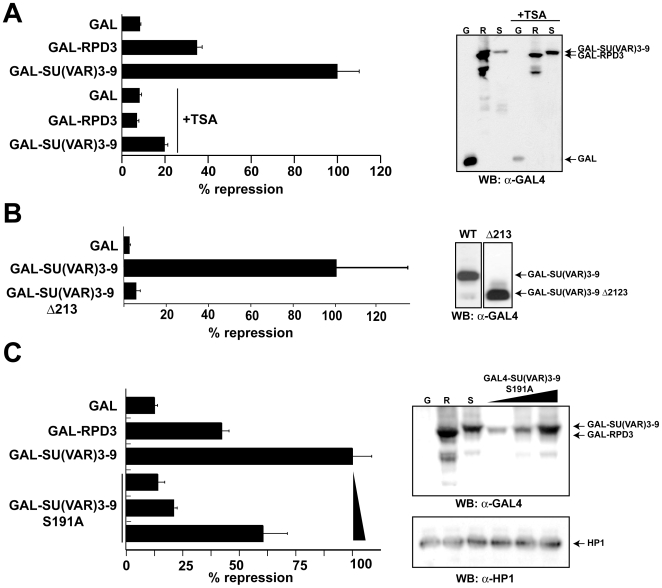
SU(VAR)3–9 can repress transcription when tethered to a promoter. (**A**) SU(VAR)3–9 represses transcription in a TSA-dependent manner. (**B**) An N-terminal truncation of SU(VAR)3–9 lacking the domain that interacts with JIL-1 and RPD3 can no longer repress transcription. (**C**) SU(VAR)3–9's transcriptional repression capacity is 2 fold reduced in a S191A mutated SU(VAR)3–9. *Drosophila* SL2 cells were co-transfected with expression constructs for dorsal and twist and the reporter construct pG_5_DE_5_-tkluc together with the indicated plasmids coding for: GAL4-DBD or GAL4 fusion proteins of SU(VAR)3–9 (and deletions/mutations thereof) and RPD3 in the presence or absence of the histone deacetylase inhibitor trichostatin A (TSA). *left:* luciferase assay of the activated reporter gene after transfection with the indicated plasmids. The repression capacity of GAL4-SU(VAR)3–9 was set to 100% and all other values were normalized accordingly. *right*: Western Blot analysis of the extracts used for the luciferase assay using an antibody specific against the GAL4-DBD [G: GAL4 DBD; R: GAL4-Rpd3; S: GAL4- SU(VAR)3–9].

To analyze if the phosphorylation had an effect on the repressive function of SU(VAR)3–9, we used an activated luciferase reporter system [35] where the repression is mediated by a GAL4-SU(VAR)3–9 fusion protein. We observed a strong transcriptional repression by SU(VAR)3–9 (Figure 4), which depended on the N-terminus of SU(VAR)3–9. A deletion that is still able to methylate H3 *in vitro* [31] fails to repress transcription (Figure 4B). This repressive effect is to a large extent mediated by an HDAC activity that is recruited to the reporter construct, as we observed a reduced repression in the presence of an HDAC inhibitor. This is in agreement with our previous results that show a genetic interaction between RPD3 and SU(VAR)3–9 [43] and by observations made in human tissue culture cells [44]. As we mapped the interaction domain between SU(VAR)3–9 and RPD3 to the N-terminus of SU(VAR)3–9 (Figure 1B) we wondered whether the transcriptional repression mutation resulted in an approximately 2-fold reduction of repression at equivalent expression levels (Figure 4C). However, the major repressive function appears to be mediated by the recruitment of an HDAC activity (Figure 4A).

### The phosphorylation of SU(VAR)3–9 *in vivo* is dependent on JIL-1

**Figure 5:**
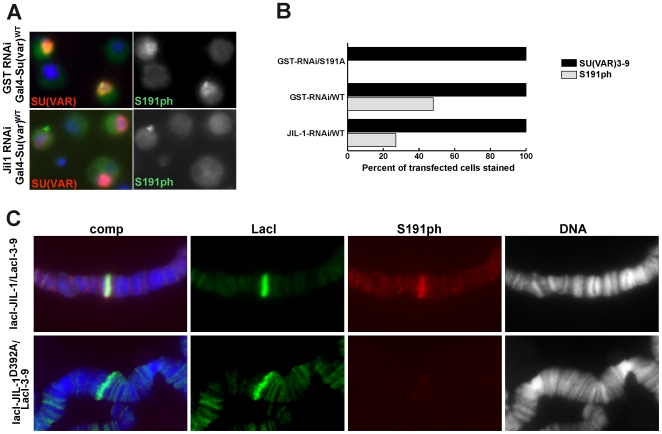
SU(VAR)3–9 is phosphorylated by JIL-1 *in vivo*. (**A**) SL2 cells were subjected to either control RNAi (GST) or JIL-1 RNAi and transfected with Gal4-SU(VAR)3–9wt or Gal4-SU(VAR)S191A. Monoclonal antibodies specific for SU(VAR)3–9 detect the protein only in the transfected cells. S191ph signal is associated with the overexpressed Gal4-SU(VAR)3–9 in the control GST RNAi but not after JIL-1 RNAi. (**B**) Quantification of 2 independent experiments showed that no S191ph signal is associated with Gal4-SU(VAR)S191A overexpressing cells (n = 192). 48% of the cells expressing Gal4-SU(VAR)wt have a S191ph signal in GST control RNAi (n = 303), and only 27% after JIL-1 RNAi (n = 311). (**C**) Phosphorylation of SU(VAR)3–9 by co-tethering of lacI-SU(VAR)3–9 and lacI-JIL-1 fusion proteins. The panels represent triple labelings of polytene squash preparations from third instar larvae homozygous for the *lacO* repeat line P11.3. LacI-SU(VAR)3–9 and LacI-JIL-1 were tethered to the *lacO* repeats in the upper panel and LacI-SU(VAR)3–9 with a catalytically inactive LacI-JIL-1 in the lower panel. LacI antibody labeling is shown in green, 3–9ph antibody labeling in red, and Hoechst labeling of DNA in blue or grey. The white arrows indicate the *lacO* repeat insertion site. *Top*: At the *lacO* repeat insertion site where LacI-SU(VAR)3–9 and LacI-JIL-1 are co-tethered there is robust phosphorylation of LacI-SU(VAR)3–9 as detected by the S191ph antibody. *Bottom*: In contrast, when LacI-SU(VAR)3–9 is co-tethered with an inactive LacI-JIL-1 construct there is no or little phosphorylation of LacI-SU(VAR)3–9 detectable by the S191ph antibody. Note: In additon to the *lacO* tethering site the LacI constructs also localize to their endogenous chromatin binding sites as shown by the localization of LacI-JIL-1 to interband regions.

To investigate if SU(VAR)3–9 is phosphorylated by JIL-1 in *Drosophila* cells as well as in flies we used the phospho-specific antibody to stain SL2 cells in which endogenous JIL-1 had been depleted by RNA interference (RNAi) and were subsequently transfected with an expression construct for GAL-SU(VAR)3–9 (Figure 5A). In control cells (treated with a control dsRNA containing the GST sequence) 48% of all cells that stained positive for GAL4-SU(VAR)3–9 also showed an immunofluorescence signal with the S191ph antibody (Figure 5B). Upon RNA interference against JIL-1 the fraction of phosphorylated SU(VAR)3–9 dropped to 27% suggesting that although JIL-1 is a major kinase for this site other kinases may also phosphorylate the N-terminus of SU(VAR)3–9. When the S191A mutant was expressed no signal was detected using the S191ph antibody, further demonstrating the specificity of the antibody (Figure 5B). Finally we wanted to know if SU(VAR)3–9 was phosphorylated by JIL-1 when both proteins are co-tethered to an ectopic binding site *in vivo*. Towards this end we used a fly strain that carried multiple *lacO* tandem repeat binding sites for the LacI repressor stably integrated on the third chromosome as described in [36]. We generated transgenic fly lines that expressed both SU(VAR)3–9 and JIL-1 LacI fusion proteins to ensure that both proteins were targeted to the *lacO* repeats. Staining polytene chromosomes of these larvae with the S191ph antibody we observed a strong labelling at the tethering site (Figure 5C). The phosphorylation of SU(VAR)3–9 at this locus was dependent on a functional JIL-1 kinase, because the targeting of an inactive JIL-1 enzyme greatly reduced the labelling by the S191ph antibody (Figure 5C).

## Discussion

In this work we describe the isolation and characterisation of a novel interactor for SU(VAR)3–9. Using the N-terminal dimerization domain as a bait we identified the chromosomal kinase JIL-1 as a SU(VAR)3–9 binding protein. The direct protein-protein interaction is mediated by the C-terminus of JIL-1. A deletion of this interaction domain in JIL-1 leads to a suppression of position effect variegation in flies [27], suggesting that the interaction between the two factors influences heterochromatic spreading. This interaction leads to a phosphorylation of SU(VAR)3–9 by JIL-1 at position S191 *in vitro* and *in vivo*. We did not observe an increase in HMT activity after phosphorylation of SU(VAR), which is consistent with the fact that a mutation of *JIL-1* does not alter global H3K9 methylation [30]. In transiently transfected reporter assays we see a strong influence of SU(VAR)3–9's N-terminus on transcriptional repression, which is mainly dependent on the recruitment of a histone deacetylase activity and is only moderately affected by the JIL-1 mediated phosphorylation. Our results suggest that the N-terminus of SU(VAR)3–9 may serve as a platform that integrates several incoming signals via multiple interactions.

A null mutation of *JIL-1* leads to a global disruption of chromatin structure [28], [29]. This has been mainly attributed to a lack of H3S10 phosphorylation and an extensive spreading of heterochromatin in the absence of JIL-1. SU(VAR)3–9 spreads to euchromatic regions in a *JIL-1^z2/z2^* homozygous mutant background [42] placing *Su(var)3–9* and *JIL-1* in the same genetic pathway. The defects of chromosome morphology that can be seen in homozygous *JIL-1^z2/z2^* mutant larvae can at least partly be rescued by crossing *JIL-1* mutant flies to a *Su(var)3–9* hypomorphic genetic background [30]. This effect is not observed in an HP1 mutant background (*Su(var)2–5*), which suggests that it is independent of the classical heterchromatin assembly. SU(VAR)3–9 or its mammalian orthologs interact with multiple other proteins [43], [44], [45], [46], [47], [48], [49], [50] in many cases via the N-terminus. Many of these interactions are important for the function of SU(VAR)3–9 as transcriptional repressor outside of its classical role in establishing heterochromatin. At the same time, the N-terminus of *Drosophila* as well as mammalian SU(VAR)3–9 serves as a heterochromatin-targeting module [51], [52], which suggests that this domain plays a central role in regulating SU(VAR)3–9's function *in vivo*.

The N-terminus of the mammalian ortholog, SUV39H1, has been shown to be phosphorylated in mammalian cells [15] but neither the kinase nor the function of the phosphorylation was determined. Our finding that this crucial domain is phosphorylated *in vitro* and *in vivo* by an enzyme that interacts genetically with *Su(var)3–9* [27], [30] provides a possible molecular explanation for the genetic interaction. However, we neither observed a strong effect on the HMT activity of the enzyme nor did we see a strong reduction in SU(VAR)3–9's ability to repress transcription in a transient reporter assay. This may reflect the current model according to which the interaction between JIL-1 and SU(VAR)3–9 plays a role only at distinct genomic loci such as the boundary regions between heterochromatin and euchromatin [27]. At such specific regions the phosphorylation and/or the interaction may change the affinity of the SU(VAR)3–9 N-terminus for its specific interactors thereby fine-tuning the molecular composition of SU(VAR)3–9 containing complexes. It will be interesting to further study several SU(VAR)3–9 interactors and investigate how the phosphorylation regulates their binding to the SU(VAR)3–9 N-terminus.
